# Heterologous expression of the filarial nematode *alt *gene products reveals their potential to inhibit immune function

**DOI:** 10.1186/1741-7007-3-8

**Published:** 2005-03-23

**Authors:** Natalia Gomez-Escobar, Clare Bennett, Lidia Prieto-Lafuente, Toni Aebischer, Clare C Blackburn, Rick M Maizels

**Affiliations:** 1 Institute of Immunology and Infection Research, University of Edinburgh, UK; 2 Institute for Stem Cell Research, University of Edinburgh, UK; 3 Max-Planck-Institut für Infektionsbiologie, Berlin, Germany

## Abstract

**Background:**

Parasites exploit sophisticated strategies to evade host immunity that require both adaptation of existing genes and evolution of new gene families. We have addressed this question by testing the immunological function of novel genes from helminth parasites, in which conventional transgenesis is not yet possible. We investigated two such novel genes from *Brugia malayi *termed *abundant larval transcript (alt)*, expression of which reaches ~5% of total transcript at the time parasites enter the human host.

**Results:**

To test the hypothesis that ALT proteins modulate host immunity, we adopted an alternative transfection strategy to express these products in the protozoan parasite *Leishmania mexicana*. We then followed the course of infection *in vitro *in macrophages and *in vivo *in mice. Expression of ALT proteins, but not a truncated mutant, conferred greater infectivity of macrophages *in vitro*, reaching 3-fold higher parasite densities. alt-transfected parasites also caused accelerated disease *in vivo*, and fewer mice were able to clear infection of organisms expressing ALT. *alt*-transfected parasites were more resistant to IFN-γ-induced killing by macrophages. Expression profiling of macrophages infected with transgenic *L. mexicana *revealed consistently higher levels of GATA-3 and SOCS-1 transcripts, both associated with the Th2-type response observed in *in vivo *filarial infection.

**Conclusion:**

*Leishmania *transfection is a tractable and informative approach to determining immunological functions of single genes from heterologous organisms. In the case of the filarial ALT proteins, our data suggest that they may participate in the Th2 bias observed in the response to parasite infection by modulating cytokine-induced signalling within immune system cells.

## Background

Pathogens have evolved many ingenious mechanisms to manipulate innate and adaptive host immune responses [1-6]. The nematode parasite *Brugia malayi *is a causative agent of the disease lymphatic filariasis, which afflicts over 100 million people in tropical countries. Mosquito-borne infective stage larvae gain entry to the human body during a blood meal, and establish long-lived infections characterised by down-regulation of host T cell and macrophage reactivity [7,8]. We have studied the profile of genes expressed in infective larvae and reported that ~5% of the mRNA transcripts from this stage correspond to two closely related genes, which we have named *abundant larval transcript (alt) -1 and -2 *[9,10]. The two genes encode proteins with 79% amino acid identity, but no similarity to any gene of known function. *alt*-like genes are present in other filarial nematode species [11,12] and are characterised by a signal peptide, a variable acidic domain, and a conserved, cysteine-rich domain. A distantly-related gene is also present in the genomes of the free-living nematodes *Caenorhabditis elegans *and *C. briggsae *but in both cases the acidic domain is absent (Gregory, Maizels and Blaxter, unpublished observation).

ALT proteins are stockpiled in the oesophageal glands of infective larvae [12] and are secreted by the parasites when they encounter mammalian culture conditions. Thus, their function may be to promote parasite survival within the host physiological or immunological environment. For example, ALTs may interfere with the critical first interaction between the innate immune system and the nematode invaders. It has been established that larval stages rapidly elicit a strong Th2 response in mice [13] and induce host macrophages to adopt a counter-inflammatory phenotype [14]. Although ALT antigens are not expressed on the parasite surface, they can induce protective immunity in animal models [9,15,16], indicating that neutralization of ALT function may be sufficient to protect the host from infection.

Transgenesis and targeted gene deletion have yet to be established for parasitic helminths, so it is not possible to investigate the biological role of ALT proteins by conventional reverse genetics. We reasoned, however, that if ALT function is fulfilled within the host rather than within the parasite, we can validly study these proteins by transgenic expression in a more tractable carrier species. We chose to test this approach with the protozoal parasite *Leishmania*, several species of which are infective to laboratory mice. *Leishmania *can readily be modified genetically [17-20] to yield lines expressing high levels of exogenous transgenes. We selected *L. mexicana *as it establishes infections in murine macrophages *in vitro*, providing experimental access to a key cell type known to be modified in filarial infection [14,21-23]. *L. mexicana *will also infect mice, and although the immunological factors determining resistance and susceptibility are not as well-defined as in *L. major *[24-26], it offers the advantage of slower *in vivo *kinetics and consequently is anticipated to be more sensitive to altering factors. Using this model, we show here that transgenic *L. mexicana *expressing the ALT proteins are more virulent in macrophages *in vitro*, and that this property is abolished by deletion of the filarial-specific acidic domain. We also show that mice infected with *alt*-transgenic *L. mexicana *harbour higher parasite burdens than controls. By studying the responses of macrophages infected with transgenic parasites, we suggest that the ALT products modulate cytokine-induced signalling and render parasites more resistant to IFN-γ-induced killing.

## Results

### *Expression of *B. malayi *ALTs in *L. mexicana

**Figure 1:**
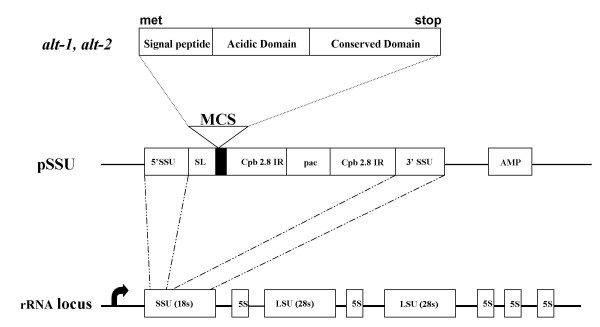
**Maps of the constructs used for homologous recombination**. Central map shows the structure of the linearized pSSU vector. 5'SSU and 3'SSU correspond to the sequences required for homologous recombination into an 18s rRNA gene; SL refers to a sequence containing a splice acceptor site; MCS to the multiple cloning site; pac to the puromycin gene and CPB-2.8 IR to the intergenic region of the CPB 2.8 gene of *L. mexicana*. The top map shows the structure of *alt-1 *and *alt-2 *transcripts that were introduced into the MCS of pSSU. The bottom map shows putative intergration into a rRNA locus. The arrow shows the start of transcription from the rRNA genes.

**Figure 2:**
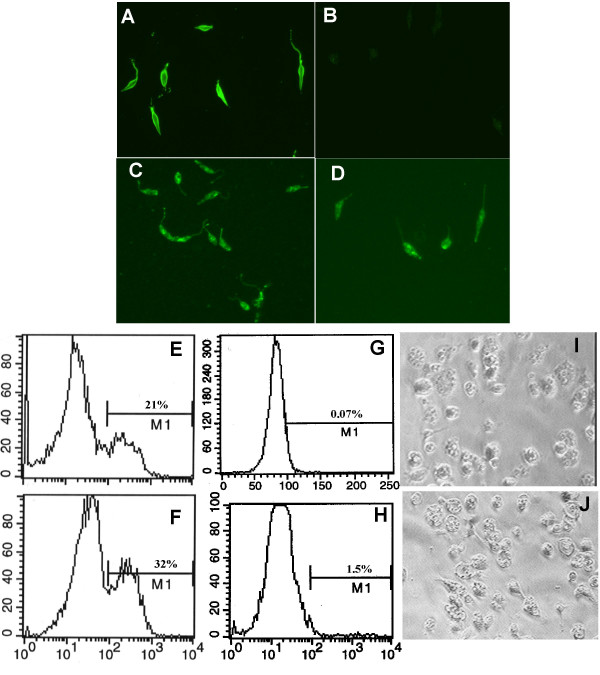
**Expression of ALT proteins in *L. mexicana *cultured promastigotes**. **(A-D) **Immunofluorescent detection of transgenic protein. Paraformaldehyde-fixed wild-type promastigotes were incubated with **(A) **anti-*L. mexicana *serum or **(B) **anti-ALT-1 antibody. **(C) **Cloned *alt-1 *transfected and **(D) ***alt-2 *transfected fixed parasites were incubated with an anti-ALT-1 antibody, which binds to both ALT-1 and ALT-2, and detected with an anti-mouse-FITC secondary antibody. **(E-H) **FACS analysis of transfected promastigotes of *L. mexicana*. Fixed promastigotes were stained, without permeabilization, with anti-ALT-1 antibody and analysed by FACS. **(E) ***alt-1*-transfected *L. mexicana*; **(F) ***alt-2*-transfected *L. mexicana*; **(G) **wild-type *L. mexicana*. Positive cells are those within the gate (M1) set above the threshold defined by reactivity of transfected parasites stained with normal mouse serum **(H)**. **(I, J) **Multiplication of transfectants within macrophages. **(I) ***In vitro*-transformed wild-type amastigotes or **(J) ***alt-1 *transfected amastigotes were added to bone marrow-derived macrophages from CBA mice at a ratio of 10 parasites per macrophage. After 24 hours at 34°C the infected macrophages were observed by microscopy.

*Bm-alt-1 *and *Bm*-*alt-2 *genes were subcloned in their entirety, including endogenous signal peptide sequences, into the recombination vector pSSU, which contains flanking sequences homologous to the 18S small subunit (SSU) rRNA locus [19,27] (Figure 1). Electroporation of *L. mexicana *was undertaken, permitting homologous recombination of the *alt-1 *and *-2 *sequences downstream of the strong polymerase I promoter into the sequence for the small sub-unit rRNA, which is known to be transcribed in both stages of the life cycle of the parasite. Following puromycin selection, multiple transgenic lines were isolated for each *alt *gene, from which representative clones were selected containing the correct insert at an appropriate integration site. Transgenic ALT expression in the free-living culture promastigote stage was confirmed in both lines by Western blot (data not shown) and immunofluorescent staining (Figure 2A–D) of permeabilized parasites with murine anti-ALT antibodies. Not only were ALT proteins found widely distributed in the transgenic protozoa, but staining of the membrane-rich flagella indicated surface expression in the promastigote stage. This was confirmed by flow cytometric analysis of anti-ALT-stained non-permeabilized transgenic promastigotes (Figure 2E, F; negative controls panels G, H).

### In vitro infection of macrophages with transfected amastigotes

**Figure 3:**
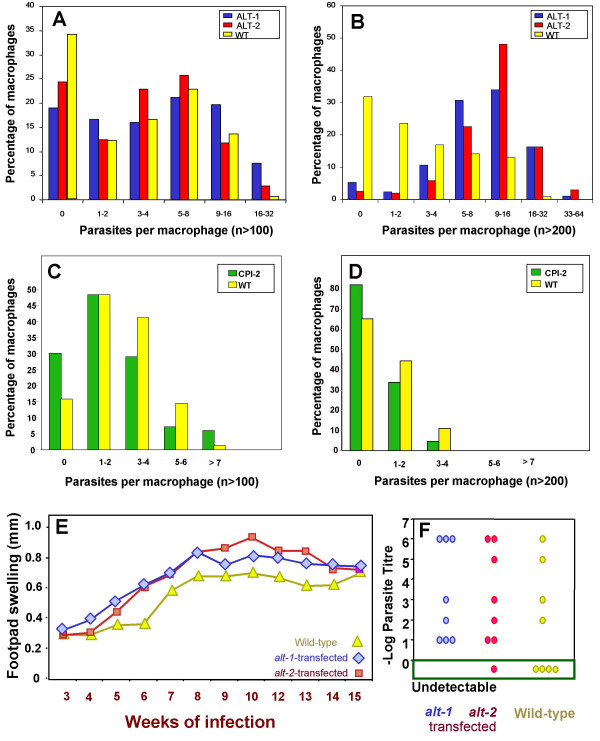
***In vitro *infection of macrophages with transfected amastigotes**. **(A, B). **Bone marrow derived macrophages from CBA mice were infected for **(A) **24 hours or **(B) **7 days with wild-type or transfected amastigotes at a ratio of 10 parasites per macrophage. Parasites were detected and counted by immunofluorescence. Between 100 and 200 macrophages were counted for each time-point. The χ^2 ^test showed significant differences in the numbers of infected vs uninfected macrophages between wild-type and either *alt-1 *or *alt-2 *transfectants at both time points (p < 0.001). **(C, D). ***In vitro *infection of macrophages with *L. mexicana *expressing the cysteine protease inhibitor-2 (CPI-2) gene of *B. malayi ***(C) **after 24 hours and **(D) **after 7 days. (**E, F**) Infection of C57BL/6 mice with transfected *L. mexicana*. **(E) **Time-course of lesion development in mice. Groups of 8 female C57BL/6 mice were injected subcutaneously in the footpad with 3 × 10^6 ^stationary-phase wild-type *L. mexicana*, *alt-1 *transfectants or *alt-2 *transfectants. Lesion size was measured weekly during the course of infection with a dial micrometer and expressed as the difference in size between the infected footpad and the contralateral uninfected footpad. One of two replicate experiments with similar results is shown; because data are not normally distributed (see for example recovery of wild-type parasites in panel F), standard error values cannot be applied. **(F) **Recovery of parasites from footpads at 15 weeks post-infection. The number of parasites in the footpad was estimated by limiting dilution assay. Results are expressed as log_10 _of the highest dilution containing parasites.

Bone-marrow-derived macrophages were infected with axenic transformed amastigotes of each line. Both wild-type (Figure 2I) and *alt*-transfected parasites (Figure 2J) were fully infective to macrophages, and continued to express transgene-encoded protein reactive by immunofluorescence (data not shown). Within 24 hours, ALT-1 and ALT-2 expressing lines were able to infect significantly more host cells (Figure 3A, p < 0.001), a contrast that was sharply accentuated by day 7 of culture (Figure 3B). Moreover, >90% of macrophages infected with *alt*-transgenic *L. mexicana *harboured at least 3 parasites, compared to <50% of cells infected with the wild-type, and overall there was a 3-fold difference in mean parasite load per macrophage. This enhancement was manifest in both *alt-1 *and *alt-2 *transgenic lines and did not affect the total number of macrophages surviving through the culture period. In contrast, transfectants expressing an unrelated filarial gene, the cystatin *Bm*-CPI-2 [28], have no effect on survival of *L. mexicana *in murine macrophages (Figure 3C, D). Thus, the observed effect is gene-dependent and is not an attribute of the transfection system.

### *Enhanced virulence of ALT-expressing *L. mexicana *promastigotes*

Analysis of transgene function in the more complex setting of *in vivo *infection was also performed, in order to test more stringently whether ALT products interfere with immunity. In two experiments, the *alt*-transfectants elicited larger lesions more quickly than either the parental wild-type strain of *L. mexicana *(Figure 3E), or a transfectant encoding GFP (not shown). In both experiments, the effect of *alt *transfection was to accelerate lesion development to a plateau after 8–10 weeks, while control parasites reached similar levels only after 12–15 weeks. Recovery of parasites from the footpads of infected mice also showed marked exacerbation: parasites were detected in our assay in 94% (15/16) of animals given transfected *L. mexicana *compared to 50% (4/8) of those receiving wild-type parasites (p = 0.03, χ^2 ^test) (Figure 3F).

### Nitric oxide production and susceptibility to NO-mediated killing

**Figure 4:**
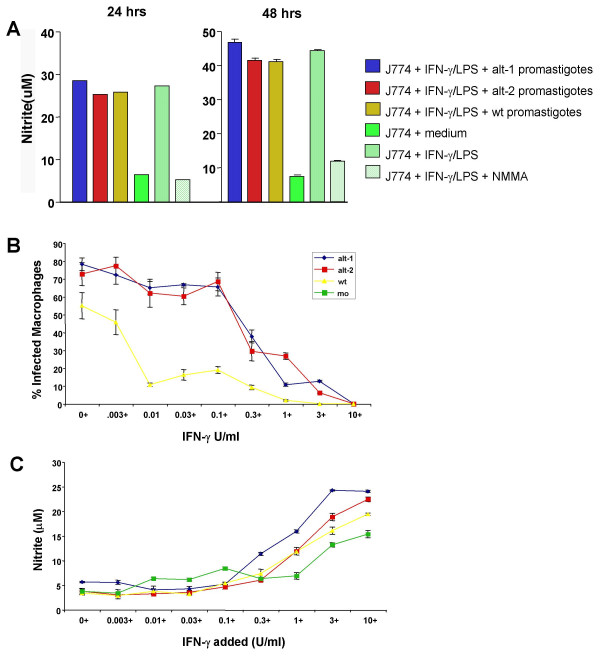
**Nitric oxide production and susceptibility to NO-mediated killing**. **A. **Nitrite production in the presence of ALT expression. J774 cells were incubated with medium alone (-ve), or stimulated with IFN-γ (40 U/ml) and LPS (10 ng/ml) (+ve) with or without the arginine analogue N-monomethyl-D-arginine (D-NMMA, 1 mM), or pre-incubated with *L. mexicana *promastigotes for 4 h before stimulation. Nitrite accumulation in the medium over the subsequent 24 h or 48 h was used as an indicator of NO production and was assayed by the Griess reaction. Each bar represents mean ± standard deviation. **B. **Survival of transfected *Leishmania *in activated macrophages. Bone marrow-derived macrophages stimulated with 100 ng/ml LPS and the indicated concentrations of IFN-γ were infected with stationary phase promastigotes of wild-type *L. mexicana *or transfected *alt-1 *and *alt-2 *parasites at a ratio of 10 parasites per cell. After 72 h at 37°C the cells were stained with Giemsa. The percentage of macrophages infected with parasites was determined by counting 4 samples of 100 cells. Each bar represents mean ± standard error of the mean. **C. **Nitrite production in response to dose of exogenous IFN-γ. Supernatants from the experiment described above (Figure 3B) were recovered and assayed by the Griess reaction for accumulated nitrite. Mo denotes uninfected macrophages. Each bar represents the mean ± standard deviation.

Because nitric oxide generation is known to be a primary factor in the control of parasite survival in macrophages [29,30], we compared levels of the NO-metabolite nitrite from J774 macrophages infected with the different parasite lines. NO production was assayed 24- and 48-hours post-infection, as the differences in parasite numbers are apparent from an early stage. Irrespective of the transgenic status of the parasite, macrophages produced near-identical amounts of NO in culture medium, measured by nitrite, at both time points (Figure 4A); thus the ALT products do not abolish the reactive nitrogen burst. To test whether there was a quantitative reduction in either NO responses or parasite sensitivity to destruction, we infected cells that had been previously stimulated with LPS and a range of doses of IFN-γ. Survival was measured by enumeration of infected macrophages 3 days later. Figure 4B shows that *alt-*transfected parasites not only achieve higher infection levels in unstimulated macrophages (no IFN-γ), but survive better in cells stimulated with intermediate IFN-γ doses (0.01–0.1 U/ml) than do wild-type organisms. Interestingly, the production of nitrite was comparable at each IFN-γ dose in all parasite types (Figure 4C), indicating that the ALT products are likely to be interfering in an NO-independent pathway of immunity.

### Gene expression in infected macrophages

**Figure 5:**
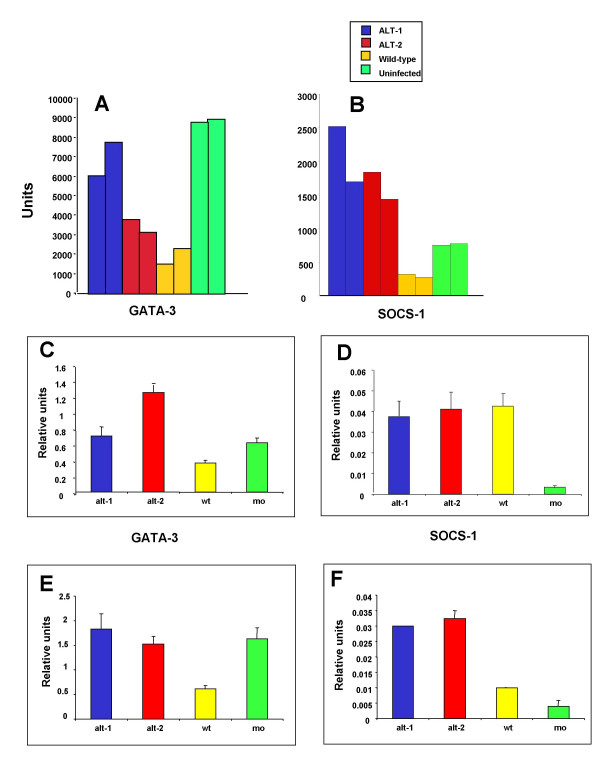
**Expression of GATA-3 and SOCS-1 in infected macrophages**. **(A,B) **Array analysis. Bone marrow-derived macrophages from CBA mice were infected for 7 days with wild-type or transfected amastigotes at a ratio of 10 parasites per macrophage. After the period of infection total RNA was extracted and used as a template to synthesise radiolabelled probes. The mouse cytokine expression array (v.1.0) from R & D Systems, on an 8 cm × 12 cm filter [56], was probed with normal macrophage cDNA, wild-type *L. mexicana*-infected macrophage cDNA, and cDNA from cells infected with *alt-1 *or *alt-2 *transfected *L. mexicana*. Quantification was carried out using a PhosphorImager and data were analyzed with ImageQuant v1.2. Results from two independent experiments are presented in adjacent bars. **(C-F) **Real-time RT-PCR analysis. Total RNA from infected and non-infected macrophages was extracted and single-stranded cDNA was synthesised. Relative quantification of the expression of the genes of interest was measured by real-time PCR using the LightCycler. The abundance of GATA-3 and SOCS-1 after 24 hours (**C **and **D**, data from n = 4 experiments), and after 7 days (**E**, n = 7, and **F**, n = 3) was expressed as a ratio of amplified product to the control, mouse S29 ribosomal protein. Each bar represents mean ± standard error of the mean. One-way analysis of variance showed that for GATA-3, both *alt-1 *and *alt-2 *transfectants were significantly higher than wild-type (p < 0.05) at both time points, and that for SOCS-1, both were significantly higher than wild-type (p < 0.01) at day 7.

To gain insight into what other mechanisms may be at play, we tested mRNA from macrophages infected with wild-type or transfected *L. mexicana *against an array of 514 genes associated with immune responsiveness formatted on the Mouse Cytokine Expression Array 1.0 (R & D Systems Inc.). Two independent experiments were performed, and hybridization of [^33^P]-labelled cDNA was measured by phosphorimaging of spots of interest. Little difference was seen in hybridization for iNOS, IL-10, IL-12 (Table 1) or many other known players in the pro- and counter-inflammatory cascades. The constancy of iNOS expression following transfection is consistent with the measurements of nitric oxide production discussed above. However, other pro-inflammatory players (TLR6, LPS-BP, LTBP3 and TNFSF11 and 12) were down-regulated relative to wild type in both *alt -1 *and *alt -2 *transfection, in replicate experiments. Moreover, substantial shifts in mRNA expression were consistently observed with certain products known to modulate the type-1/type-2 balance. Bone-marrow-derived macrophages, infected 7 days earlier with wild-type *L. mexicana *infection, showed significant ablation of GATA-3 and SOCS-1, but expression was maintained or enhanced in cells infected with *alt*-transgenic parasites (Figure 5A and 5B). These effects were reproduced with both alt genes in both experiments (Table 1).

Because array hybridization may under certain conditions be non-linear with respect to mRNA concentrations, we performed quantitative RT-PCR with GATA-3- and SOCS-1-specific primers on replicate samples of infected macrophages taken at 24 h (Figure 5C, D) and 7 days (E, F). For GATA-3, these data reinforce the conclusion that while wild-type infection results in a 2–3-fold loss of GATA-3 expression, *alt*-transgenic infected macrophages maintain the level of this transcription factor throughout the course of *in vitro *infection (Figure 5C and 5E). Interestingly, SOCS-1 is sharply upregulated by 24 h in all infected macrophages, but elevated expression remains evident only where *alt *transgenic parasites are present (Figure 5D and 5F). Although it is possible that larger parasite numbers in the *alt-*transfection system may in themselves alter macrophage gene expression, parasite densities were only slightly shifted at 24 h, when GATA-3 expression was markedly different in wildtype and transfected cell infections.

### Up-regulation of GATA-3 and SOCS-1 in filarial infection

**Figure 6:**
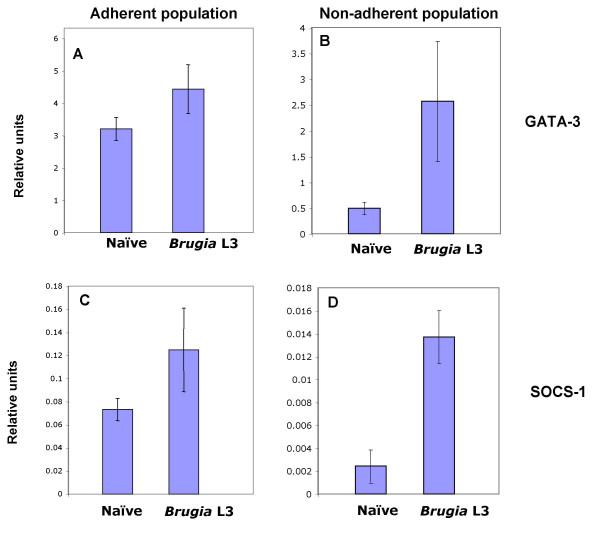
**Up-regulation of GATA-3 and SOCS-1 in filarial infection **Peritoneal cells were recovered 7 days after ip infection with 200 L3s. **(A, C) **Adherent (macrophage-enriched) and **(B, D) **non-adherent (predominantly lymphocytes) populations were separated and cDNA synthesised from total RNA. The abundance of GATA-3 **(A, B) **and SOCS-1 **(C, D) **was determined as in Figure 5. Each bar represents mean ± standard error of the mean. (One way analysis of variance; p = 0.05). Data are representative of two experiments.

We then tested whether GATA-3 and SOCS-1 are up-regulated by live infection with *B. malayi *larvae *in vivo*. Mice were injected i.p with 200 larvae, and 7 days later peritoneal cell populations were recovered by lavage with medium. We then performed real time PCR on an adherent macrophage enriched cell population and a non-adherent cell population, predominantly lymphocytes. As shown in Figure 6A and 6C, the macrophage-rich adherent population showed a modest increase in both GATA-3 and SOCS-1 expression, whereas a very substantial rise in both was seen in the non-adherent cells (Figure 6B and 6D). It seems that *B. malayi *infection results in up-regulation of the same genes as observed with ALT in macrophages, in both macrophage and non-macrophage subsets.

### The acidic domain of ALT is essential for biological function

**Figure 7:**
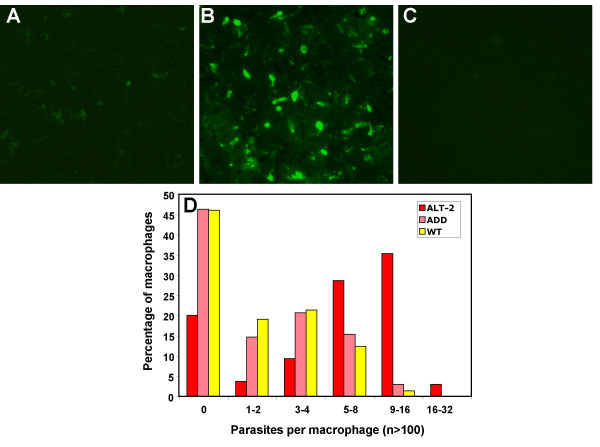
**Deletion of the Acidic Domain abolishes the functional effect of ALT transfection. (A-C) **Immunofluorescent detection of ADD (acidic domain deleted mutant of ALT-2) protein. Paraformaldehyde-fixed wild-type promastigotes **(A) **and cloned *ADD *transfected parasites **(B) **were incubated with mouse antiserum to anti-ALT-2, and detected with an anti-mouse-FITC secondary antibody. **(C) **Cloned *ADD *transfected parasites were also incubated with normal mouse serum. **(D) **Bone marrow-derived macrophages were infected with stationary phase amastigotes of wild-type *L. mexicana *or transfected *alt-2 *and *ADD *parasites at a ratio of 10 parasites per cell. After 7 days, cells were stained with Giemsa. The percentage of macrophages infected with parasites was determined by counting 3 samples (from 3 different wells) of 100 cells. The χ^2 ^test showed significant differences in the number of infected macrophages between wild-type and *alt-2 *transfectants (p < 0.001), but not between wild-type and *add*-transfected parasites.

The enhanced virulence phenotype conferred by *alt *transfection, measured by *in vitro *infectivity to macrophages, provides a ready and tractable system for submolecular analysis of functional domains of the ALT protein. We investigated whether the *N*-terminal acidic domain, which is unique to the filarial genes, was important for the immunological activity of the protein. We constructed a truncated mutant of ALT-2, named *acidic domain deleted (add)*, which was cloned into the pSSU vector and electroporated into *L. mexicana*. These transfectants expressed immuno-reactive protein (Figure 7A–C) and were able to infect bone marrow-derived macrophages. However, *add*-transfected parasites did not display the greater infectivity seen with *alt*-transfectants, but showed a phenotype indistinguishable from wild-type parasites (Figure 7D). Thus, the acidic domain is required for functional expression of the immunological effects of the ALT proteins. Similar analyses will permit the future definition of the critical residues required for immunomodulatory activity.

## Discussion

Long-lived helminth parasites have evolved highly effective strategies to evade host immunity, requiring both adaptation of existing genes and evolution of new gene families [6]. With genomes that encode many thousands of proteins, these parasites are likely to be repositories of numerous novel 'immune evasion genes' with no or only weak sequence similarity to known products [31]. The imminent completion of genome sequence information for major helminth parasites [32-34] accentuates the problem of how to identify functional immune modulators among numerous novel gene sequences. We hypothesized that transcripts for secreted immunomodulatory proteins would be among the most abundant mRNAs at key points in the parasite life cycle. Two such genes are those that encode the abundant larval transcripts (ALT) proteins, which are released by larval parasites ready to infect the mammalian host and represent 5% of the total mRNA at this stage [9].

To test whether the ALT proteins are functional immune evasion products, we transfected each *alt *gene into *L. mexicana *and showed that infection of macrophages *in vitro *is exacerbated by expression of either ALT protein. Moreover, mice infected with *alt*-transgenic parasites display more rapid lesion development and higher parasite burdens than controls. Our results also demonstrate that *alt*-transfected parasites are more resistant to INF-γ-induced killing by macrophages, supporting our hypothesis that ALT proteins act to modify host immune responses in filarial infection. These data validate the transfection strategy in general and, in highlighting changes in key intracellular factors resulting from ALT expression, justify the selection of a protozoal carrier to test the function of a helminth gene.

A further advantage of the system we describe here is the facility with which selected mutants can be analysed with an *in vitro *read-out of infectivity to macrophages. We constructed a deletion mutant lacking the N-terminal acidic domain, which shows most variation between filarial species and is not present in distantly related genes from free-living nematode organisms. This deletion showed a clear-cut abolition of the *alt *phenotype, indicating the essential functional nature of this sequence and paving the way for a finer analysis of structure-function relationships in a tractable experimental system.

We have also extended the use of this transfection system to the analysis of other parasite genes that are hypothesised to be immunomodulatory. For example, filarial parasites produce homologues of mammalian macrophage migration inhibitory factor, MIF [39,40], a cytokine generally considered to be an acute pro-inflammatory agent [41,42]. It is, however, paradoxical that long-lived tissue pathogens produce a potentially inflammatory mediator, and we therefore used the *Leishmania *transfection system to test the hypothesis that long-term MIF production may promote parasite survival. *L. mexicana *organisms transfected with *B. malayi *MIF homologues were tested first *in vitro*, in which setting they were less infective to macrophages, rarely exceeding one parasite per host cell. This result was consistent with an immediate pro-inflammatory action of MIF on macrophages. However, when tested *in vivo *over a 4- or 8-week-period, MIF-transfected parasites were able to survive better than wild-type, indicating that over the longer term, filarial MIF homologues are able to exert a down-modulatory effect on host immunity (Prieto-Lafuente, manuscript in preparation). We are now using this system to analyse the gene expression profiles of macrophages infected with MIF-transfected parasites in both *in vitro *and *in vivo *settings.

These data illustrate the facility with which the *Leishmania *transfection system can be used in parallel for *in vitro *and *in vivo *experiments, and the importance of an *in vivo *read-out to assay gene effects within the immune system as a whole. In addition, our work has demonstrated that *Leishmania *transfection for helminth gene analysis is equally applicable for two completely unrelated, but immunologically important, gene families.

The application of this new strategy to transfection of *L. mexicana *is ideally suited to the study of macrophage modulation by genes predicted to function in this environment. Parallel investigations can also be envisaged by transfection of *L. major*, which will permit a more thorough analysis of T cell modulation to be undertaken. For example, resistance to *L. major *is clearly associated with development of a parasite-specific Th1 response, and it is possible that parasite genes that inhibit Th1 differentiation will alter the course of *L. major *infection *in vivo*. These studies are now under way.

Macrophages are known to be a critical cell population in the immune response to filarial parasites at successive points in time. First, they participate in innate defences against invading larvae [35,36]; second, if infection becomes established, they evolve an IL-4-dependent "alternatively activated" phenotype, which is broadly immunosuppressive [8,37]; and third, in late stage infection, they clear the bloodstream of microfilarial forms through a nitric oxide-dependent pathway [21,38]. Thus, the reduction in IFN-γ-responsiveness in macrophages harbouring *alt*-transfected parasites has resonance for initial host susceptibility, and longer-term propensity for chronic infection, as well as the ability to eliminate the blood-borne Mf stage.

A key outcome of the present study is that ALT expression is associated with up-regulation of GATA-3 and SOCS-1. GATA-3 is a pivotal transcription factor for the development and function of the Th2 pathway [43,44] and has not previously been reported in mouse macrophages; hence, at the present time, downstream genes activated by GATA-3 in macrophages have not been defined. Significantly, GATA-3 is required for embryonic development and has recently been shown to be essential for the differentiation [45] and effector function [46] of murine eosinophils. Thus, GATA-3 expression in macrophages may fulfil an important role, possibly in conjunction with the altered phenotype of these cells in chronic parasite infections [8,47].

SOCS-1 is a member of the Suppressor Of Cytokine Signalling family of proteins, which regulate signal transduction by IFN-γ and structurally related cytokines [48,49]. SOCS-1 is particularly important in macrophage responsiveness to inflammatory stimuli [50], countermanding IFN-γ by directly inhibiting the JAK kinase associated with the IFN-γ receptor [51]. SOCS-1 is known to inhibit the IFN-γ-dependent killing of *Leishmania *parasites, because macrophages from SOCS-1 null mice require 100-fold less IFN-γ to clear infection [52]. Indeed, in the absence of SOCS-1, there is generalised activation of the immune system, causing autoimmune pathology [53,54]. Thus, by up-regulating SOCS-1, cells expressing ALT may have down-shifted responsiveness to inflammatory cytokines, requiring exogenous stimulation to induce *Leishmania *killing (Figure 5B). In the context of *Brugia *infection, SOCS-1 induction by ALT proteins could explain why macrophages fail to develop the IFN-γ-activated phenotype, and instead express a counter-inflammatory profile [8].

Overall, these data suggest that the ALT proteins play a role in the evasion strategy of the filarial nematode, by directly amplifying Th2 responses and/or interfering with signals necessary for the development of pro-inflammatory Th1 populations. It is interesting to note that *in vivo *exposure to live infective larvae of *B. malayi *induces a prompt Th2 response measurable by 24 h and increasing to 10 days post-infection [13]. We have repeated these experiments by intraperitoneal inoculation of live L3 and find up-regulation of GATA-3 and SOCS-1 in both adherent and non-adherent peritoneal cells (Figure 6).

## Conclusion

The novel approach we have described permits, for the first time, the elucidation of gene function for a major group of biologically and medically important parasites, which in the example presented here provides non-intuitive results linking parasite secretions to host cell signalling. The transfection strategy will also accommodate a mutagenesis analysis of structure-function relationships in unique gene families. In conclusion, our system provides a solution to one of the major obstacles facing helminth parasite immunology in the post-genomic era and offers a fascinating insight into the molecular and cellular intricacies of pathogen manipulation of host immune responsiveness.

## Methods

### Mice and parasites

Six- to eight-week old female C57BL/6 and CBA mice were used. *L. mexicana *(strain MNYC/BZ/62/M379) promastigotes were cultured *in vitro *in semi-defined medium/10% heat inactivated foetal calf serum (hiFCS)/1% penicillin-streptomycin (complete SDM) at 26°C. Amastigotes were cultured axenically at 34°C in Schneider's Drosophila medium (Gibco BLR) supplemented with 20% hiFCS and 3.9 g/l 2-(N-morpholino)ethanesulfonic acid (Sigma, U.K.). Transgenic parasites were cultured under the same conditions with the addition of 20 μg/ml puromycin (Sigma, U.K).

### Cell culture

The murine macrophage cell line J774 was passaged in DMEM containing 10% hiFCS/1% penicillin-streptomycin/1% L-glutamine and cultured at 37°C in 5% CO_2_.

### *Leishmania expression construct for *B. malayi *ALTs*

Primers were designed to amplify the entire coding region of *alt-1 *and *alt-2 *from a *B. malayi *L3 cDNA library. The oligonucleotides used for *alt-1 *were 5'-CCGCTCGAG**ATG**AACAAATTGCTAATAGCA-3' (sense, initiating codon in bold) and 5'-TGCTCTAGA**TTA**CGAGCATTGCCAACTTTC-3' (antisense, terminating codon in bold); and for *alt-2*, 5'-CCGCTCGAG**ATG**AATAAACTTTTAATAGCA-3' (sense) and 5'-TGCTCTAGAC**TAT**GCGCATT GCCAACCTGC-3' (antisense). After an initial denaturation step at 95°C for 5 min the PCR was cycled between 94, 55 and 72°C (1 min each) for 35 rounds, followed by 1 round at 72°C for 10 min. The fragments were digested with *XhoI *and *XbaI *and cloned into the pSSU vector (13), yielding pSSU-*alt-1 *and pSSU-*alt-2*. Clones were fully sequenced on both strands. DNA was extracted from pSSU-*alt-1 *and pSSU-*alt-2 *using the Qiagen Miniprep kit following the manufacturer's instructions

### Site directed mutagenesis

An ALT-*2 *mutant, in which the acidic domain of the protein (amino acids 24–49) were deleted, was generated using the Exite PCR-based site-directed mutagenesis kit (Stratagene, USA) following the manufacturer's instructions. The pSSU-*alt-2 *construct (see above) was used as the template in a PCR reaction containing two oligonucleotides: 5'-TGATTCTGATACACACGGGAGTGT-3' (antisense, primer phosphorylated) and 5'-TATGTAACCAAAGGGAATTTGTT-3' (sense). The cycling parameters were as follows: 1 cycle of 1 min at 94°C, 4 min at 53°C and 2 min at 72°C; followed by 10 cycles of 1 min at 94°C, 2 min at 55°C (adding 10 s after each cycle) and 1 min at 72°C; and a final cycle of 5 min at 72°C. After the PCR the nonmutated parental plasmids were digested with *Dpn *restriction enzyme. The undigested linear DNA was then polished with *Pfu *DNA polymerase and ligated at 37°C for 1 h with T4 DNA ligase. The ligated DNA was then transformed into *E. coli*, yielding a pSSU-ADD (acidic domain deleted) construct. The insert was sequenced to verify that the intended mutation was correctly constructed.

### *Transfection of *Leishmania

Logarithmic phase promastigotes (4 × 10^7^) were electroporated with 10 μg of *PmeI *linearized fragments of either pSSU-*alt-1*, pSSU-*alt-2 *or pSSU-*add*. Clones were selected on 24-well plates in complete SDM supplemented with 20 μg/ml of puromycin and further propagated in a culture volume of 10 ml.

### Immunofluorescence

Promastigotes were washed in PBS, fixed in 2% paraformaldehyde for 30 min at room temperature, and quenched in NH_4_Cl for 10 min. Wild-type parasites were incubated with dilution buffer (0.1% saponin, 2% goat serum) in the presence of anti-*L*. *mexicana *rabbit serum or anti-ALT mouse serum (from C57BL/6 mice) for 45 min. Following 3 washes with wash buffer (0.1% saponin, PBS) the parasites were incubated with either an anti-rabbit-FITC or an anti-mouse-FITC secondary antibody (DAKO, Denmark) for 45 min. They were then washed 3 times before mounting with Cityfluor (Cityfluor Ltd, UK) for microscopy.

### Flow cytometry

Promastigotes were washed in PBS, resuspended at 1 × 10^6^/ml and fixed without permeabilization in 2% paraformaldehyde for 15 min at room temperature. After two washes in PBS, 10% FCS (FACS wash), the parasites were stained with anti-ALT-1 antibody or normal mouse serum diluted in FACS wash. This was followed by incubation with a FITC anti-mouse secondary antibody (DAKO, Denmark). Flow cytometric analysis was performed using a FACScan (Becton Dickinson) and analysed using CellQuest 3.1 software.

### Preparation of bone marrow-derived macrophages (BMM)

BMM were obtained from femurs and tibias of 6- to 8-week old CBA mice by flushing the bones with DMEM (Gibco, BLR) containing 10% hiFCS/1% penicillin-streptomycin/1% L-glutamine. Cells were centrifuged and plated out in non-tissue culture Petri dishes at a density of 5 × 10^5^/ml in complete DMEM, supplemented with 20% (v/v) L929 cell-conditioned medium as a source of M-CSF and 20% hiFCS. After 6 days at 37°C, the cells were detached by incubation in PBS containing 3 mM EDTA and 10 mM glucose, plated and cultured in small non-tissue culture Petri dishes at 2.5 × 10^5^/ml in complete DMEM for 24 h at 37°C.

### *Infection of BMM with *L. mexicana

Day 7 BMM were infected for 24 h or 7 days at 34°C with wild-type or *alt*-transfected amastigotes at a ratio of 10 parasites per macrophage. After the period of infection macrophages were harvested as described above, washed with PBS and fixed and permeabilized using a "Fix & Perm kit" (Pharmigen); 1 × 10^5 ^cells were centrifuged on to glass slides with a Cytospin. Intracellular Parasites were detected and counted by immunofluorescence as described above. Between 100 and 200 macrophages were counted for each time-point.

### *Infections *in vivo

For *L. mexicana in vivo *infections, groups of 8 female C57BL/6 mice were injected subcutaneously in the footpad with 3 × 10^6 ^stationary-phase wild-type *L. mexicana*, *alt-1 *transfectants and *alt-2 *transfectants. Lesion size was measured weekly during the course of infection with a dial micrometer and expressed as the difference in size between the infected footpad and the contralateral uninfected footpad.

For *B. malayi in vivo *infections, groups of 5 female C57BL/6 mice were injected intraperitoneally with 200 infective larvae recovered from crushed *Aedes aegypti *mosquitoes. After the experimental period the mice were euthanized by terminal anaesthetic, and peritoneal cells (PEC) were harvested by thorough washing of the peritoneal cavity with 15 ml of ice-cold RPMI supplemented with 10% FCS. The harvested PEC were plated in 24-well culture plates at 2 × 10^6 ^cells/well. Following 3 h at 37°C to allow cells to adhere, both the non-adherent and the adherent macrophage-enriched cell populations were harvested.

### Parasite quantification

The number of parasites in the footpad was estimated by limiting dilution assay. Infected footpads were harvested in cold PBS after removal of the skin. Footpad tissue was dispersed through a cell strainer and resuspended in PBS-1% penicillin/streptomycin. After centrifugation the pellet was resuspended in complete SDM. The cell suspension was then serially diluted in 10-fold steps, in quadruplicate, in 96-well plates. The plates were incubated for 4 days at 27°C; the wells were then observed for parasite growth.

### Measurement of nitrite production

J774 macrophages were plated out on 96-well plates (1 × 10^5^/well) and incubated at 37°C for 24 h. The medium was then removed, the cells were washed twice, and *L. mexicana *promastigotes were added for 4 h at a ratio of 10 parasites per well. IFN-γ (40 U/ml) and LPS (10 ng/ml) were then added, with or without the arginine analogue *N*-monomethyl-D-arginine (D-NMMA), 1 mM. Nitrite accumulation in medium over the subsequent 24–48 h was used as an indicator of NO production and was assayed by the Griess reaction in which 100 μl of Griess reagent [55] was added to 100 μl of each supernatant in triplicate wells in a 96-well plate. Plates were read at 490 nm against reference wavelength 620 nm using an ELISA plate reader. NaNO_2 _was used to make a standard curve for each plate reading.

### Leishmanicidal assay

BMM (5 × 10^4^) were plated out on glass coverslips in 24-well plates, allowed to adhere for 24 h, and stimulated with 100 ng/ml LPS and the indicated concentrations of IFN-γ. Cells were incubated for 6 h at 37°C before adding stationary phase promastigotes of wild-type *L. mexicana *or transfected *alt-1 *and *alt-2 *parasites at a ratio of 10 parasites per cell. After 72 h at 37°C the cells were stained with Giemsa. The percentage of macrophages infected with parasites was determined by counting 4 samples of 100 cells.

### RNA isolation

Total RNA was isolated from non-infected and infected BMM as well as adherent and non-adherent cell populations using TRIzol Reagent (Invitrogen, Life Technologies) according to the manufacturer's instructions. The RNA was subjected to DNase treatment (Ambion, INC) to eliminate genomic contamination, according to the manufacturer's instructions

### Gene array analysis

Gene expression was analysed using the Mouse Cytokine Expression Array (R&D systems). The mouse cytokine-specific primers were first annealed to the total RNA, which was then reverse transcribed in the presence of SuperScript II (Invitrogen, Life Technologies) and [α-^33^P]dCTP. The radiolabelled cDNA probes generated from non-infected and infected cells were hybridized to identical membranes containing the mouse cDNA arrays. Following hybridization, high stringency washes were performed and the membranes were subjected to autoradiography. Quantification was carried out using a PhosphorImager and data analyzed with ImageQuant v1.2.

### Real Time RT-PCR analysis

Total RNA was extracted in Trizol, as described above, and single-stranded cDNA was synthesized using MMLV reverse transcriptase (Stratagene). Relative quantification of the expression of the genes of interest was measured by real-time PCR using the LightCycler (Roche Molecular Biochemicals). PCR amplifications were performed in 10 μl volumes containing 1 μl cDNA, 2.5 mM MgCl2, 3 μmM primers and the LightCycler-DNA SYBR Green I mix (Qiagen). The reaction was performed in the following conditions: 15 min activation step at 95°C for one cycle, 15 s denaturation at 95°C, 20 s annealing of primers at 50°C and 15 s elongation at 72°C, for 50 cycles. The fluorescent DNA binding dye SYBR Green was monitored after each cycle at 80°C. Five serial 1:2 dilutions of *alt-2 *infected macrophages cDNA were used to produce a standard curve in each reaction. The abundances of GATA-3 and SOCS-1 were expressed as ratios of amplified product to the control, mouse S29 ribosomal protein. Primers for RT-PCR analysis were as follows: GATA-3: 5'-CTA CGG TGC AGA GGT ATC C-3' and 5'-GAT GGA CGT CTT GGA GAA GG-3'; SOCS-1: 5'-ACC TTC TTG GTG CGC GAC AGT CGC CAA-3' and 5'-GGA ACT CAG GTA GTC ACG GAG TAC-3'; and S29 ribosomal protein: 5'-ATG GGT CAC CAG CAG CTC TAC-3' and 5'-GTC CAA CTT AAT GAA GCC TAT-3'.

## Abbreviations

ALT, abundant larval transcript protein; *alt*, abundant larval transcript gene or mRNA; BMM, bone marrow-derived macrophages; FACS, fluorescence-activated cell sorter; L3, third-stage infective larva; NO, nitric oxide.

## Authors' contributions

NG-E designed and led the experimental work, including molecular constructs, cell transfection, immunological assays, array and RT-PCR analyses. She also drafted the manuscript. CB and LP-L made constructs in pSSU, maintained Leishmania cultures, participated in electroporation procedures, macrophage infection, in vivo infection and dilution assays. TA designed the transfection system and devised the *Leishmania *transfection methodology. TA, CCB and RMM jointly conceived the transfection strategy for *Brugia *genes, critically assessed progress, and made revisions to the draft manuscript. The overall study was co-ordinated by RMM who also completed the draft of the manuscript. All authors read and approved the final manuscript.

## Figures and Tables

**Table 1:** Array hybridization intensities for 5 immune-related genes in macrophages infected with filarial alt genes.

Mouse gene	Transfectant	Experiment 1	Experiment 2
iNOS	alt-1	6847.3	2260.4
	alt-2	6951.1	2224.4
	wild-type	7105.6	2280.1
	uninfected	6807.7	2296.3

IL-10	alt-1	605.7	171.3
	alt-2	564.2	173.8
	wild-type	563.5	169.7
	uninfected	579.6	167.3

IL-12	alt-1	150.8	184.2
	alt-2	152.8	187.9
	wild-type	166.3	178.5
	uninfected	197.3	192.5

GATA-3	alt-1	6063.9	7822.8
	alt-2	3817.1	3168.4
	wild-type	1550.1	2324.6
	uninfected	8848.2	8995.3

SOCS-1	alt-1	2517.8	1695.1
	alt-2	1837.7	1437.7
	wild-type	493.1	429.6
	uninfected	752.4	791.6

Data represent Phosphorimager quantification of radioactive probe bound to R&D Systems Mouse Immunological Array v1.0. Two independent experiments are presented.
